# Systematic search of Bayesian statistics in the field of psychotraumatology

**DOI:** 10.1080/20008198.2017.1375339

**Published:** 2017-10-31

**Authors:** Rens van de Schoot, Naomi Schalken, Miranda Olff

**Affiliations:** ^a^ Department of Methods and Statistics, Utrecht University, The Netherlands; ^b^ Optentia Research Program, Faculty of Humanities, North-West University, South Africa; ^c^ Department of Psychiatry, Academic Medical Center, University of Amsterdam, The Netherlands; ^d^ Arq Psychotrauma Expert Group, Diemen, The Netherlands

## 1. Introduction

In recent years there has been increased interest in Bayesian analysis in many disciplines; for example, see the systematic reviews in the fields of educational science (König & van de Schoot, 2017), organizational science (Kruschke, 2010), psychometrics (Rupp, Dey, & Zumbo, 2004), health technology (Spiegelhalter, Myles, Jones, & Abrams, 2000), epidemiology (Rietbergen, 2017), medicine (Ashby, 2006), and psychology (Van de Schoot, Winter, Ryan, Zondervan-Zwijnenburg, & Depaoli, 2017). Also, the use of Bayesian analyses in the field of psychotraumatology was advocated during a meeting of the International Society for Traumatic Stress Studies (ISTSS) global meetings program^1^.

Bayesian methods implement Bayes’ theorem, which states that prior beliefs are updated with data, and this process produces updated beliefs about model parameters. The prior is based on how much information we believe we have preceding data collection, as well as how accurate we believe that information to be. Within Bayesian statistics, priors can come from any source; for example, a meta-analysis, a previous study or, even, expert consensus. For a basic introduction to Bayesian statistics we refer to Yalch (2016) or to Van de Schoot et al. (2014), where many references are provided for the novice as well as the more technical reader.

To further encourage the use of Bayesian statistics in the field of psychotraumatology, we initiated a special issue on the use of Bayesian statistics. Below, we first introduce briefly how Bayesian statistics is already applied by means of describing the results of a systematic search in the psychotrauma field; thereafter, we describe the papers submitted as part of this special issue on Bayesian statistics.

**Figure 1.**
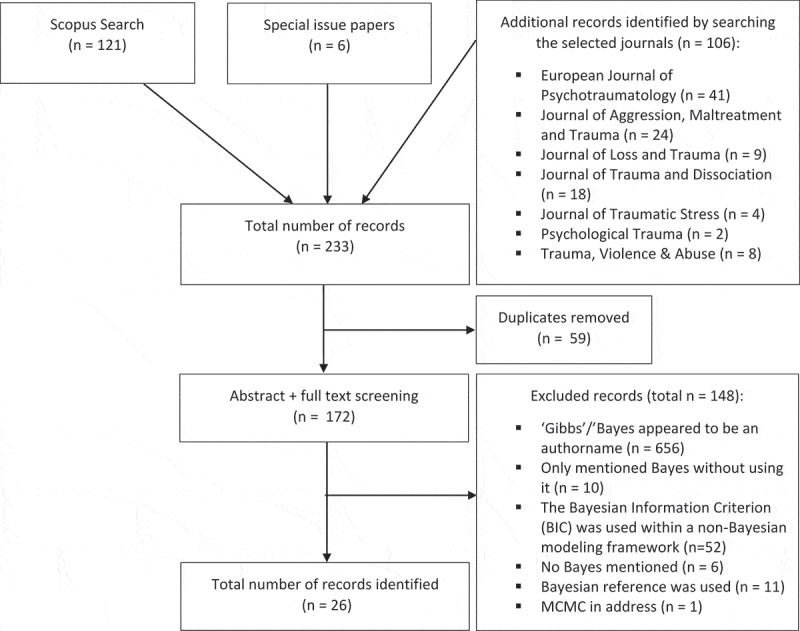
Flow diagram of the systematic search and selection process.

To describe the previously published Bayesian papers in the field of psychotraumatology, we conducted a systematic search focusing on Scopus in seven journals (Figure 1). No limitations on the time period of the search were used, which resulted in articles from 1989 to 2017. We used the following search terms: ‘Bayes*’, ‘MCMC’ (Markov chain Monte Carlo), and ‘Gibbs’. We also included six papers that were part of the special issue on Bayesian statistics, which are described in Section 3. Following the initial identification of relevant articles, exact duplicates were excluded, and another 148 papers were excluded for various reasons after reading the full text (see Figure 1 for details). The total number of empirical articles found to be eligible was 18, plus another two short papers that were part of the ISTSS global meetings program, and six as part of the special issue (total *n* = 26) (Table 1). See the online supplementary materials published on the Open Science Framework (https://osf.io/wxkpu/) for more information: our logbook, Endnote file with all the references, the exclusion decisions, and an overview of the included papers.

**Table 1.** Overview of the included papers.

Reference	Topic	Why Bayes
Allen et al. (2002)	Pathological dissociative taxon membership, absorption, and reported childhood trauma	Bayes probability
DePrince (2005)	Social cognition and revictimization risk	Bayes probability
Cuperus et al. (2016)^a^	Virtual reality paradigm and real-life trauma	Support instead of *p* values + informative hypothesis testing
De Roos et al. (2011)	CBT and EMDR	To deal with missing data
Fedina (2015)	Sex trafficking in the USA	Including experts to deal with missing data
Depaoli et al. (2015)^b^	Theory-driven solution of PTSD trajectories	Small samples
Goodman et al. (2003)	Pathological dissociation and personality diagnosis, and self-directed injury	Bayes probability
Hagenaars, Holmes, Klaassen, and Elzinga (2017)^a^	Tetris after PTSD	Support instead of *p* values + informative hypothesis testing
Krypotos et al. (2017)^a^	Bayesian analysis in threat conditioning research	Support instead of *p* values + informative hypothesis testing
Kuijer, Marshall, and Bishop (2014)	Relationship between pretrauma variables, such as neuroticism, optimism, self-control, pretrauma depression, and post-earthquake adjustment (PTSD symptoms)	Technical – non-normality
Küffer et al. (2016)^a^	Childhood adversity, dysfunctional rearing behavior, psychological health offspring	Small samples
Littel et al. (2017)^a^	EMDR	Support instead of *p* values + informative hypothesis testing
Michélsen, Therup-Svedenlöf, Backheden, and Schulman (2017)	Post-traumatic growth and depreciation	Not mentioned
Mohr and Rosén (2017)	Protective factors on post-traumatic growth	To deal with missing data
Mueller-Pfeiffer et al. (2010)	Somatoform Dissociation Questionnaire (SDQ-20)	Not mentioned
Neylan et al. (2014)	Biomarkers for combat-related PTSD	To model causal relations
Van De Schoot (2015)^b^	Latent growth mixture models to estimate PTSD trajectories	Small samples
Van De Schoot et al. (2015)	PTSD after burn event	To deal with small samples
Van Schie et al. (2016)^a^	Modification of episodic memories by novel learning	Support instead of *p* values + informative hypothesis testing
Van Schie et al. (2016)	EMDR	Support instead of *p* values + informative hypothesis testing
Van Veen et al. (2016)	EMDR	Support instead of *p* values + informative hypothesis testing
Wahlström et al. (2013)	Reporting psychological symptoms	Technical reason – dichotomous mediator
Yalch (2016)	Applicability of Bayesian statistics in psychological trauma research	Introduction to Bayes
Yalch et al. (2016)	Effects of prenatal and post-birth intimate partner violence	Technical – non-normality
Yalch and Levendosky (2014)	Betrayal trauma and dimensions of borderline personality organization	Technical – non-normality
Yalch et al. (2017)	Survivors of intimate partner violence	Technical – non-normality

**^a^**Part of the special issue.

**^b^**Part of the International Society for Traumatic Stress Studies (ISTSS) global meetings program.

CBT, cognitive behavioral therapy; EMDR, eye movement desensitization and reprocessing; PTSD, post-traumatic stress disorder.

## 2. Reasons for using Bayesian statistics

There are different reasons for using Bayesian statistics. Here, we provide a few examples of such reasons mentioned in the field of psychotraumatology.^2^

### 2.1. Flexible hypothesis testing

The first reason why Bayesian statistics is used is that it provides a flexible alternative to null hypothesis significance testing. van Veen, Engelhard, and van den Hout (2016) tested several predictions from working memory theory that could explain the efficacy of eye movement desensitization and reprocessing (EMDR). The authors translated their hypothesis into a set of specific hypotheses, and tested these informative hypotheses (Hoijtink, 2012) using Bayes factors (BFs). A similar procedure was followed by van Schie, van Veen, Engelhard, Klugkist, and van den Hout (2016), who used BFs to investigate the role of working memory capacities and eye movement speed in reducing memory vividness and emotionality. Moreover, Bayesian *t *tests are more accurate in the case of non-normal data or outlying observations, as mentioned by Yalch, Black, Martin, and Levendosky (2016), Yalch and Levendosky (2014), and Yalch, Schroder, and Dawood (2017).

### 2.2. Updating probabilities

A second reason to use Bayes is because of the updating of prior knowledge with data into the posterior, for example in the computation of taxon membership (Allen, Fultz, Huntoon, & Brethour, 2002; DePrince, 2005; Goodman et al., 2003). As explained by Goodman et al. (2003), the probability of a taxon membership can be computed based on what is known before any data are collected. That is, the investigator has a subjective probability about who can be a member of the taxon and who cannot. After data have been collected, these prior probabilities are updated with the data and provide a more accurate probability of taxon membership.

### 2.3. No need for large data sets

Another reason why Bayesian statistics is so attractive is that it does not rely on large data sets. Van De Schoot, Broere, Perryck, Zondervan-Zwijnenburg, and Van Loey (2015) showed in a simulation study, and with an actual application to a limited data set of burn survivors, that Bayes outperformed the default estimation method for structural equation models. When using maximum likelihood estimation, there appear to be power issues for small samples and therefore it becomes difficult to find meaningful results. The authors investigated what kind of solutions Bayesian estimation can provide to conduct research with small sample sizes. They concluded that Bayes is able to overcome small sample issues, but only when background information is added to the model via the so-called prior distributions (see also Depaoli, Van De Schoot, Van Loey, & Sijbrandij, 2015; Van de Schoot, 2015).

### 2.4. Imputation of missing data

A fourth advantage of Bayesian analysis is the treatment of missing data, as applied by de Roos et al. (2011), who conducted a randomized controlled trial to compare cognitive behavioral therapy with EMDR in a group of disaster-exposed children. In the intention-to-treat analysis, the authors replaced outcome data that were missing owing to dropouts using multiple imputations by fully conditional specification using the MICE package (Van Buuren & Oudshoorn, 1999). Mohr and Rosén (2017) also used this imputation strategy to deal with missing data in their research on childhood experiences of abuse and neglect and later post-traumatic growth. Fedina (2015) solved the missing data issue by using experts to specify a ‘reasonable replacement’ for missingness in their research on human trafficking. As shown in many simulation studies (e.g. Peeters, Zondervan-Zwijnenburg, Vink, & van de Schoot, 2015), Bayesian imputation of missing data outperforms many other ways of dealing with missing data, including missing data as a result of dropouts (see Asendorpf, Van De Schoot, Denissen, & Hutteman, 2014, for more details).

### 2.5. Ability to deal with technical complexities

A fourth reason is that Bayes can deal with technical issues where classical methods, such as maximum likelihood, fail or require too much computational time. This argument is used in Wahlström, Michélsen, Schulman, Backheden, and Keskinen-Rosenqvist (2013), who used a dichotomous moderating variable to examine the reporting of different physical symptoms over time in survivors of a tsunami. In Neylan, Schadt, and Yehuda (2014), Bayesian statistics was proposed as a causal modeling approach to identify factors that affect biological networks, such as environmental traumatic exposures and other biological risk factors.

## 3. Special issue

As part of the special issue, six papers have been published.

Krypotos, Klugkist, and Engelhard (2017) introduced Bayesian analysis in the field of threat conditioning, which is an important paradigm in the experimental study of post-traumatic stress disorder (PTSD). In this approach, an initially neutral stimulus is paired with an evolutionary aversive stimulus or event. Consequently, the neutral stimulus will evoke threat or fear responses. To simplify the analysis of threat conditioning data and the calculation of BFs, the authors developed the ‘condir’ package in R, including an easy-to-use Shiny application.^3^ Users merely have to provide the right conditioning data, and condir easily generates the final results in terms of BFs.

van Schie, van Veen, van den Hout, and Engelhard (2016) tested null hypotheses using BFs in the R package BayesFactor (Morey, Rouder, & Jamil, 2014) to replicate the laboratory study of Wichert, Wolf, and Schwabe (2013). When consolidated memories are reactivated, they can become unstable and sensitive to change before they are stored into long-term memory. With a mechanism such as novel learning, the reactivated memories could be disrupted. The authors were able to replicate the finding that there was memory impairment and memory updating in the group with both reactivation and new learning in comparison to the reactivation-only group and the group without reactivation or new learning. However, they failed to replicate the difference between new learning in combination with reactivation and new learning alone.

Littel, van Schie, and van den Hout (2017) used BFs to whether prior knowledge about the EMDR treatment would change the memory-attenuating effects of eye movements. The authors used the software BIEMS (Mulder, Hoijtink, & de Leeuw, 2012) to evaluate informative hypotheses specified based on background knowledge. They concluded that prior knowledge was found to have no to modest effects on eye movements.

Hagenaars, Holmes, Klaassen, and Elzinga (2017) used BFs to test a set of very specific hypotheses about a trauma film paradigm (TFP), which was used to examine the effects of a supposedly visuospatial task (the computer game Tetris) and a more verbal task (word games) versus no task (reactivation only) on intrusion frequency in a reconsolidation time frame. They found support for two hypotheses: (a) an intervention effect, with both task conditions being equally effective (reactivation + Tetris = reactivation + word games < reactivation only); and (b) a modality effect, with word games being the most effective task (reactivation + word games < reactivation + Tetris < reactivation only).

Küffer, Thoma, and Maercker (2016) investigated early-life adversity experienced by parents that had an impact on their children’s mental health. The authors tested the null hypothesis using BFs in the software JASP (Marsman & Wagenmakers, 2016). They investigated the role of parental rearing behavior in the transgenerational conveyance of aversive childhood events experienced by parents as Verding children on the mental health of their offspring. The ‘Verdingkinder’ were former Swiss indentured child laborers, for whom there was a high prevalence of adverse childhood experiences and poor mental health. The authors found substantial support for higher levels of childhood adversity in the Verding children and more problematic rearing behavior, but found no substantial mental health problems in their offspring.

Cuperus, Klaassen, Hagenaars, and Engelhard (2016) evaluated informative hypotheses using BIEMS regarding the use of virtual reality (VR) in the TFP using BFs. With the TFP, the effects of psychological trauma can be studied under controlled experimental settings. However, there is a lack of active behavioral engagement in this approach, which is a disadvantage. Therefore, as shown by the authors, the use of VR can be used as an alternative.

## 4. Conclusion

Bayesian analyses are slowly creeping into many areas of science, including the field of psychotraumatology. Even with a systematic search limited to psychotraumatology, we see an increasing number of papers using Bayesian analyses for a range of reasons, as described above. The method of evaluating a set of informative hypotheses, each representing a theoretical meaningful ordering of relevant parameters, seems especially popular. The advantage of dealing with limited data in the Bayesian framework is another probable reason why more researchers would (need to) make the switch. For whatever reason Bayesian analysis is used, the prior settings are always of importance and should be described in detail. The idea of making optimal use of existing information is an attractive and efficient way forward.

## Notes

1.
https://www.rensvandeschoot.com/agenda/symposium-estimating-ptsd-trajectories/.

2. In two articles (Michélsen et al., 2017; Mueller-Pfeiffer et al., 2010), Bayes was used but without any argumentation.

3.
https://utrecht-university.shinyapps.io/Condir/.
